# Commercially Available Apps to Support Healthy Family Meals: User Testing of App Utility, Acceptability, and Engagement

**DOI:** 10.2196/22990

**Published:** 2021-05-07

**Authors:** Chelsea E Mauch, Rachel A Laws, Ivanka Prichard, Anthony J Maeder, Thomas P Wycherley, Rebecca K Golley

**Affiliations:** 1 Caring Futures Institute College of Nursing and Health Sciences Flinders University Adelaide Australia; 2 Early Prevention of Obesity in Childhood Centre of Research Excellence Sydney Australia; 3 School of Exercise and Nutrition Science Institute for Physical Activity and Nutrition Deakin University Melbourne Australia; 4 Sport, Health, Activity, Performance and Exercise Research Centre College of Nursing and Health Sciences Flinders University Adelaide Australia; 5 Flinders Digital Health Research Centre College of Nursing and Health Sciences Flinders University Adelaide Australia; 6 Alliance for Research in Exercise, Nutrition and Activity Allied Health and Human Performance University of South Australia Adelaide Australia

**Keywords:** diet, nutrition, family, mobile applications, behavior modification

## Abstract

**Background:**

Parents juggling caregiving and paid employment encounter a range of barriers in providing healthy food to their families. Mobile apps have the potential to help parents in planning, purchasing, and preparing healthy family food. The utility and acceptability of apps for supporting parents are unknown. User perspectives of existing technology, such as commercially available apps, can guide the development of evidence-based apps in the future.

**Objective:**

This study aims to determine the feasibility of existing commercially available apps for supporting the healthy food provision practices of working parents.

**Methods:**

Working parents (N=133) were recruited via the web and completed a 10-item Capability, Opportunity, Motivation, and Behavior (COM-B) self-evaluation survey assessing their needs in relation to the provision of healthy family meals. A total of 5 apps were selected for testing, including a meal planning app, recipe app, recipe manager app, family organizer app, and barcode scanning app. Survey items were mapped to app features, with a subsample of parents (67/133, 50.4%) allocated 2 apps each to trial simultaneously over 4 weeks. A semistructured interview exploring app utility and acceptability and a web-based survey, including the System Usability Scale and the user version of the Mobile App Rating Scale, followed app testing. The interview data were analyzed using a theoretical thematic approach.

**Results:**

Survey participants (N=133; mean age 34 years, SD 4 years) were mainly mothers (130/133, 97.7%) and partnered (122/133, 91.7%). Participants identified a need for healthy recipes (109/133, 82% agreed or strongly agreed) and time for food provision processes (107/133, 80.5%). Engagement quality was the lowest rated domain of the user version of the Mobile App Rating Scale across all 5 apps (mean score per app ranging from 3.0 to 3.7 out of a maximum of 5). The family organizer, requiring a high level of user input, was rated the lowest for usability (median 48, IQR 34-73). In the interviews, participants weighed the benefits of the apps (ie, time saving) against the effort involved in using them in determining their acceptability. Organization was a subtheme emerging from interviews, associated with the use of meal planners and shopping lists. Meal planners and shopping lists were used in time, while behavior was occurring.

**Conclusions:**

Meal planning apps and features promoting organization present feasible, time-saving solutions to support healthy food provision practices. Attention must be paid to enhancing app automation and integration, as well as recipe and nutrition content, to ensure that apps do not add to the time burden of food provision and are supportive of healthy food provision behavior in time.

## Introduction

### Background

Suboptimal dietary intake is a major public health concern because of its role in the development of noncommunicable diseases (NCDs) [1]. In 2016, NCDs were responsible for 70% of deaths worldwide [2]. Key dietary risk factors for NCDs include inadequate intake of vegetables, fruit, and wholegrains and excessive intake of energy-dense, nutrient-poor foods, also termed discretionary choices [1]. A total of 90% of Australian adults do not meet the recommended daily serves of vegetables, and more than a third of daily energy intake is from discretionary choices [3]. Similar trends have been observed internationally [4,5]. Poor dietary patterns start young and persist over time. Australian children’s diet quality mirrors adult patterns by 4-8 years of age [3,6]. Supporting parents to provide healthy food to themselves and their families will improve population diet quality.

There has been a trend toward greater female workforce participation in modern households [7,8]. In Australia in 2019, 70% of mothers in dual-parent households were working, whereas 60% of mothers in single-parent households were working [7]. Parents juggling caregiving and paid employment experience a range of barriers in providing healthy food to their families. The Capability, Opportunity, Motivation, and Behavior (COM-B) system [9] describes 3 key conditions that interact to enable a behavior to occur: capability, opportunity, and motivation. Parent-focused nutrition interventions to date have tended to target capability (eg, knowledge and skills) and motivation (eg, confidence in supporting child health) [10]. However, opportunity-related enablers, such as adequate time for food provision, are important and promote resilience against the broader unhealthy food environment [11,12]. Therefore, it is important to consider a range of enablers relevant to the planning, purchasing, and preparation of food in the development of future nutrition interventions.

The time- and staff-intensive nature of traditional face-to-face interventions make them impractical in a resource-scarce health promotion environment. Mobile apps offer advantages over face-to-face interventions, such as the delivery of interventions in everyday situations [13]. A review identified 51 commercially available apps that addressed the planning, purchasing, and preparation of food [14]. The review found that meal planning, family organizer, and recipe manager apps incorporated features promoting organization that could address potential barriers to healthy meal provision, such as time scarcity and cognitive load [14]. However, app content generally mapped to relatively few behavior change techniques and was not targeted toward healthy eating in a family context [14].

### Objectives

The next step in understanding the behavioral potential of these types of apps and features in a family food provision context is to gain insights from target users. User perspectives can inform the design of evidence-based apps that are informed by user context and needs [15-17]. This study sought user perspectives on commercially available apps to inform future app development or refinement [18]. This study aims to determine the feasibility of existing commercially available apps and app features for supporting healthy food provision practices in working parents by exploring the following:

1. The utility of apps and app features to support planning, purchasing, and preparation of food
2. The acceptability of apps and app features in terms of quality, usability, functionality, and engagement.

## Methods

### Study Design

This feasibility study was conducted between February and June 2019 using a mixed methods design. Participants completed a baseline survey, with a subsample undertaking a 4-week app testing period, followed by another survey and semistructured interview. A total of 5 apps were selected for testing based on a previous review of commercially available apps [14]. Selected apps represented the key content and features of interest identified in the previous review (Multimedia Appendix 1 [14]). They rated well for quality compared with similar apps, were available in a free or freemium format, and were available on Apple and Android operating systems. Only one of the apps tested has published research available regarding its development [19].

### Study Sample and Recruitment

Eligibility criteria included being a single or partnered parent in paid employment, with themselves or their partner having returned to work from a period of parental leave in the last 6 months. Other eligibility criteria included being based in Australia and the main food gatekeeper of the household. Individuals who did not own an Apple or Android mobile device with internet access or whose partner was not in paid employment were excluded.

Recruitment was conducted via Facebook and flyers posted around a university campus and in childcare centers. These recruitment channels have been used successfully in previous research [20-22]. Baseline survey completion constituted consent for the survey only. Participants provided contact details at the end of the survey to indicate their interest in app testing. Consent for app testing was by return email, with reminders sent to nonresponders until recruitment and app allocation goals were met. A target sample size of 50 was set for app testing, with at least 10 participants testing each app. This was comparable with similar feasibility and pilot app testing studies [22-24]. Ethics approval was provided by the Flinders University Social and Behavioral Research Ethics Committee (approval no. 8211).

### App Testing

A total of 10 baseline questions modeled on the COM-B self-evaluation survey [25] exploring the perceived enablers of healthy food provision were mapped to the apps for testing (Multimedia Appendix 1). Participants were assigned 2 of the 5 mobile apps for testing, based on their responses to the baseline questions. This allowed the allocation of apps based on need. The allocation of 2 apps allowed participants to envisage how complementary content and features could be combined. Consenting participants were contacted via telephone or email for app allocation and setup and emailed a checklist of tasks to prompt use of a range of app features (eg, viewing a recipe, creating a new meal plan, and setting a day and time to receive meal planning reminders). They were encouraged to use both the apps as little or as much as they wished during the following 4 weeks.

### Follow-up

At the completion of the 4-week app testing period, participants were emailed a link to the follow-up survey, with 1 to 3 reminder emails sent to nonresponders. Following receipt of the follow-up survey, participants were contacted by telephone to conduct a semistructured interview (until data saturation was reached). Participants involved in app testing were provided with a meal kit or grocery voucher to the value of Aus $85 (US $66) in compensation for their time.

### Data Collection

#### Baseline Survey

Demographic survey items included parents’ age, sex, highest level of education, relationship status, household income, and work hours; partner’s work hours (if applicable); and the number and age of children in the household. Diet quality measures were adapted from the validated Short Food Survey [26] and included questions relating to fruits and vegetables (2 items) and discretionary choice intake (10 items). For discretionary choice items, frequency of consumption (ie, daily, weekly, and monthly) was followed by a question regarding the number of times it was usually consumed (eg, twice or thrice). Discretionary choice items included sugar-sweetened beverages, takeaway foods, fried potatoes, savory snacks, savory pastries, sweet baked goods, snack bars, confectionaries, and frozen desserts.

The 10 COM-B self-evaluation items (Multimedia Appendix 2) were rated on a 7-point Likert scale ranging from strongly disagree (1) to strongly agree (7) [25]. Scores from the 2 items mapping to each app were summed, with participants allocated the 2 apps receiving the highest aggregate score. Where an app was not allocated to at least 10 participants, some participants were allocated these apps despite a lower COM-B score, to ensure that adequate data were collected for each app.

#### Follow-up Survey

Frequency of app use was measured in the follow-up survey for each of the 4 weeks using a 4-point response scale (ie, *didn’t use the app*, *once*, *2-4 times*, and *5 or more times*). The duration of app use was measured using a 3-point response scale (ie, *less than 1 min*, *1-5 min*, and *more than 5 min*). The System Usability Scale (SUS), a brief scale of 10 statements covering the complexity or ease of use of apps, was used to assess usability [27,28]. Participants indicated their agreement on a 5-point scale ranging from strongly disagree (1) to strongly agree (5). User-perceived app quality was measured using the user version of the Mobile App Rating Scale (uMARS) [29]. The 16-items comprising the engagement, functionality, esthetics, and subjective quality subscales were included in this study [29]. The information quality subscale was replaced with an item regarding app credibility, as the included apps contained minimal information, and credibility has been shown to be important to app engagement [21].

#### Semistructured Interviews

The Consolidated Criteria for Reporting Qualitative Research checklist guided the presentation of qualitative methods and findings [30]. Semistructured interviews were conducted by a female research assistant with qualitative research experience and a research focus on family meals. The research assistant had no previous contact with participants. Three of the interviews were conducted by CEM, including a pilot interview and 2 final interviews. Interviews took between 30 and 60 minutes and were audio recorded with the participants’ permission, using a speaker phone and audio recorder. Interviews were transcribed verbatim by an independent company.

Interview questions addressed the acceptability of app features and content, user engagement with the apps, the usefulness of the apps in addressing food provision, improvements required, and general suggestions for future app development. Questions were repeated for each of the 2 apps. The questions were tested with a research assistant and piloted with one participant. As interviews were conducted, CEM listened to the audio and discussed progress with the research assistant. Once data saturation for an app or app combination was reached, determined by no new information emerging, participants testing those apps were only asked to complete the follow-up survey. Single parents and parents of a lower income were prioritized for interviews to represent as diverse a sample as possible.

### Data Analysis

#### Quantitative Data

Quantitative data were analyzed using SPSS version 22 (IBM Corporation). Parental work hours were converted from continuous variables into groups (ie, part time=1 to <35 hours per week and full time=35+ hours) and combined to describe the family work schedule. Discretionary items were summed as total serves per day using age-and sex-specific adjustment factors [31]. Demographic data, diet quality, and COM-B self-evaluation items were presented descriptively (eg, n [%], mean [SD], and median [IQR] for COM-B items due to a positive skew in the data).

Follow-up data regarding self-reported frequency and duration of app use were calculated for each app and presented descriptively as n (%). SUS scores were converted to a score out of 100 [27], with the median (IQR) score of the sample presented, due to a positive skew in the data. A median score below 50 was indicative of poor app usability, 50 to 70 was marginal, more than 70 was passable, and more than 90 was superior [28]. Scores for uMARS items were summed and averaged for each subscale and across all items for the overall uMARS score.

#### Qualitative Data

Transcriptions were coded using NVivo (QSR International) and analyzed using a theoretical thematic approach [32]. Coding took an inductive approach, with interview data initially sorted into groups based on the study objectives, interview questions, and app characteristics. Coding was conducted by CEM, who organized the data into major and minor themes and generated an initial conceptual model [32]. A meeting between coauthors was undertaken to discuss and refine themes, after which the final conceptual model with links back to quantitative data was ascertained. When presenting the interview results, names were changed to preserve anonymity.

## Results

### Sample Characteristics

**Figure 1:**
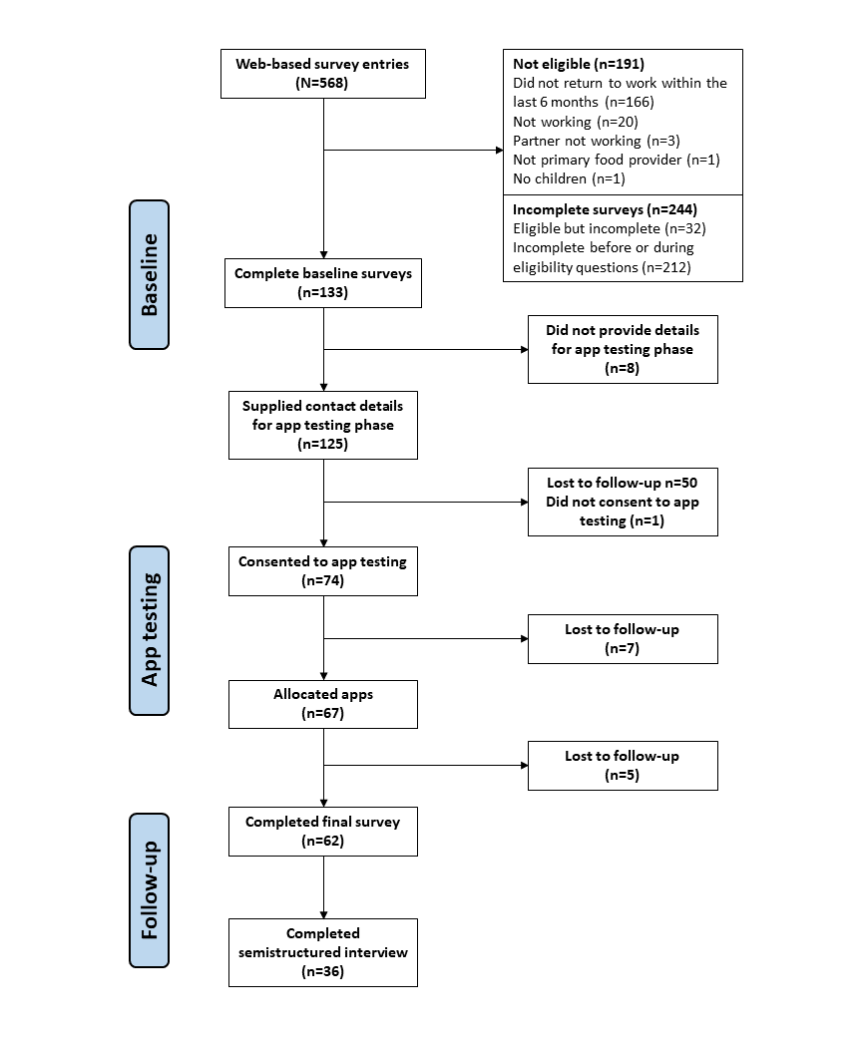
Flow of participants through the study.

Figure 1 describes participant flow throughout the study. Participants completing the baseline survey (N=133) were mostly partnered (122/133, 91.7%) and females (130/133, 97.7%). Two-thirds (90/133, 67.7%) of households included one full-time working parent and one part-time working parent. In most households (106/133, 79.7%), the age of the youngest child was less than 2 years, and 60.2% (80/133) included more than one child. Only 6.8% (9/133) of participants met the Australian guidelines for vegetable intake, whereas most (107/133, 80.5%) met the guidelines for fruit intake. Participants reported consuming 3.0 (SD 2.1) discretionary choice serves per day, excluding alcohol (Table 1). Compared with the baseline sample, participants who completed interviews were more likely to have a university degree (28/36, 78% vs 83/133, 62.4%), be unpartnered (5/36, 14% vs 11/133, 8.3%), and have an income below Aus $70,000 (US $54,221) per annum (9/36, 25% vs 25/133, 18.7%; Table 1).

Table 2 presents the results of the COM-B self-evaluation items used to allocate apps. More than three-quarters of participants suggested a need for healthy recipes and meal ideas (109/133, 82% agreed or strongly agreed; median 6, IQR 6-7) and time to plan, buy, and prepare healthy meals (107/133, 80.5%; median 6, IQR 6-7). Almost two-thirds suggested a need for a better way of planning and recording meals and groceries (87/133, 65.4%; median 6, IQR 5-6), whereas food selection and cooking skills were not high priorities for this sample.

**Table 1:** Demographic characteristics of the survey sample at baseline and the sample included in the qualitative analysis.

Characteristics			Baseline survey data sample^a^		Interview subsample^b^
Age (years) mean (SD)			33.8 (4.3)		33.6 (4.3)
**Sex, n (%)**					
	Female	130 (97.7)		35 (97.2)	
	Male	3 (2.3)		1 (2.8)	
**Highest level of education, n (%)**					
	University	83 (62.4)		28 (77.8)	
	No university	50 (37.6)		8 (22.2)	
**Relationship status, n (%)**					
	Partnered	122 (91.7)		31 (86.1)	
	Single	11 (8.3)		5 (13.9)	
**Number of children, n (%)**					
	One	50 (38.5)		13 (37.1)	
	More than one	80 (61.5)		22 (62.9)	
**Age of youngest child (years), n (%)**					
	Less than 2	106 (79.7)		29 (80.6)	
	2-4	21 (15.8)		5 (13.9)	
	5-12	6 (4.5)		2 (5.6)	
**Household income (gross per annum), n (%)**					
	Less than Aus $70,000 (US $54,221)	25 (18.8)		9 (25.0)	
	Aus $70,000 (US $54,221) or more	92 (69.2)		24 (66.7)	
	Prefer not to say	16 (12.0)		3 (8.3)	
**Family work schedule, n (%)**					
	Both part time	9 (6.8)		1 (2.8)	
	Part time and full time	90 (67.7)		26 (72.2)	
	Both full time	23 (17.3)		4 (11.1)	
	Single working parent	11 (8.3)		5 (13.9)	
**Vegetable intake (serves per day), n (%)**					
	1 or less	26 (19.5)		8 (22.2)	
	2-4	98 (73.7)		26 (72.2)	
	5 or more	9 (6.8)		2 (5.6)	
**Fruit intake (serves per day), n (%)**					
	Do not eat fruit	4 (3.0)		1 (2.8)	
	1 or less	69 (51.9)		17 (47.2)	
	2 or more	60 (45.1)		18 (50.0)	
Discretionary intake (serves)^c^, mean (SD)			3.0 (2.1)		3.0 (2.2)

^a^Samples range from 118 to 133 due to missing data.

^b^Subsamples range from 33 to 36 due to missing data.

^c^Excluding alcohol.

**Table 2:** COM-B self-evaluation item mean (SD) scores and proportions of the sample responding agreed or strongly agreed (N=133).

COM-B^a^ domain and item			Item score^b^, median (IQR)		Agreed or strongly agreed^c^, n (%)
**Capability**					
	Have better food preparation or cooking skills	5 (3-6)		44 (33.1)	
	Learn how to choose healthy food at the supermarket	5 (2-5)		30 (22.6)	
	Learn how to plan healthy meals	6 (5-6)		72 (54.1)	
**Opportunity**					
	Have more time to plan, buy, and prepare healthy meals	6 (6-7)		107 (80.5)	
	Have more healthy recipes and meal ideas	6 (6-7)		109 (82.0)	
	Have guidance in choosing healthy food and meals	5 (4-6)		38 (28.6)	
	Have a better way of planning and recording meals and groceries for the coming week	6 (5-6)		87 (65.4)	
	Have more support or help from my partner or family	5 (4-6)		43 (32.3)	
	Have more reminders to plan, shop or cook	5 (4-6)		43 (32.3)	
**Motivation**					
	Have clear goals or plans toward preparing healthy meals	6 (5-6)		76 (57.1)	

^a^COM-B: Capability, Opportunity, Motivation, and Behavior.

^b^1=strongly disagree to 7=strongly agree.

^c^Score of 6 or 7 (agreed or strongly agreed).

### Follow-up and Interview Data

**Figure 2:**
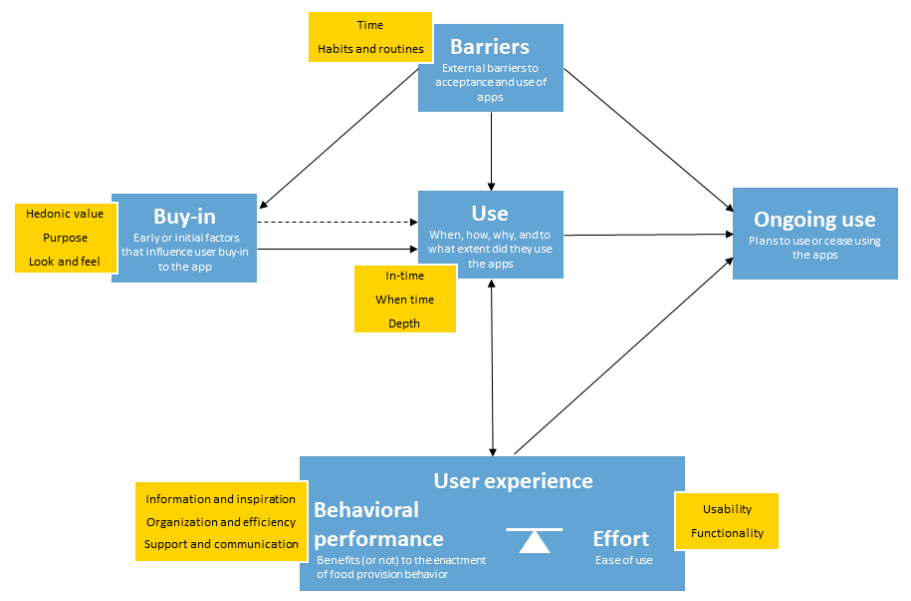
Conceptual diagram of major themes (blue) and minor themes (yellow) and how these may relate to the ongoing use or disengagement with the apps.

Of the 67 participants who were allocated apps, 62 (93%) completed the follow-up survey and 36 (54%) participants completed the interviews (Figure 1). Among those completing interviews, 9 different combinations of apps were allocated. The most common sets of apps allocated were the recipe manager and family organizer (n=7) or meal planning app (n=6) and the barcode scanning and recipe app (n=6). Figure 2 demonstrates the conceptual model of major and minor themes emerging from the semistructured interviews, and Table 3 provides examples of quotes relating to each subtheme.

**Table 3:** Participants’ perspectives of app utility and acceptability and their engagement with apps: major and minor themes and illustrative quotes.

Major and minor themes			Illustrative quotes
**Buy-in**			
	Purpose	“(...) for probably what I was looking for which was meal planning, [meal planning app] was more appropriate.” (Mia, working 1 to <21 hours per week) “...I think, it had a big overarching purpose but lots of, like, little purposes in there that just, kind of meant that you had to wade through more stuff to figure out what you wanted to use it for.” (Jo, working 21 to <35 hours per week)	
	Hedonic value	“...I hadn’t even, um, really thought about the fact that there were apps out there to support with meal prep and healthy eating and all of that...” (Harper, working 21 to <35 hours per week) “...I didn’t want to use the app. (...) I wasn’t excited by it.” (Sophie, working 21 to <35 hours per week)	
	Look and feel	“...you’d like trust and you feel comfort in knowing that, you know, it just feels like a team of people has worked behind it...” (Tiffany, working 21 to <35 hours per week)	
**Use**			
	In-time	“So we would (...) decide on the meals (...) then I would go grocery shopping (...) then I would put it away until I needed to cook every night.” (Blair, working 21 to <35 hours per week)	
	When-time	“...when the kids were sort of asleep (...) just looked up some recipes and that type of thing so—‘cause I just had some time to actually do it.” (Dianne, working 1 to <21 hours per week)	
	Depth	“I don’t think I was even aware of it. (...) I don’t think I even found that function.” (Bianca, working 21 to <35 hours per week)	
**Barriers**			
	Time	“I think, real or just perceived, I think, that’s a, um, a time issue, I feel, like, (...) there’s other things I should be doing...” (Cora, working 21 to <35 hours per week)	
	Habits and routines	“...the app can be as brilliant as it is but if I’m not going to actually actively go out of my way to build that habit, (...) it’s only as good as I’m going to make it.” (Sophie, working 21 to <35 hours per week) “...I actually ended up changing jobs, like, right in the middle of, um, trialing the app. (...) I tried to settle back to just doing what was easy...” (Harper, working 21 to <35 hours per week)	
**User experience** **—** **behavioral performance**			
	Information and instructions	“...if I was, you know, tired or whatever, I might just turn to a freezer meal (...) whereas this kind of made me think, like making it from scratch and like, still find easy ways to get, you know, vegetables into my kids” (Kathryn, working 1 to <21 hours per week) “...aligned to the Australian (...) food guidelines (...) helping me, um, tick off how many serves I’m getting in each meal, something like that would be a nice bonus.” (Blair, working 21 to <35 hours per week)	
	Organization and efficiency	“...having the plan there it, sort of, I mean, it almost makes you more accountable (...) it really cuts out the excuses of, oh, I’m tired, we’re running late, let’s get a pizza.” (Cora, working 21 to <35 hours per week) “Like, it took away the decisions, decisions I had to make, I think, I had already made them, and then, I didn’t need to stress about it, basically.” (Blair, working 21 to <35 hours per week) “Here’s the recipe (...) adjust your shopping list (...) go through the, your supermarket of choice, get it delivered when you want, done.” (Fae, working 21 to <35 hours per week)	
	Support and communication	“...it would definitely be accessible across multiple devices. Um, so, you know, that, like, that everyone who’s old enough and interested, in the family could contribute. (...) he wouldn’t be constantly asking me every day, ‘What’s for tea tonight?’” (Brianna, working 35+ hours per week)	
**User experience** **—** **effort**			
	Usability	“It’s quick for meals and then the grocery, and then it comes up with a list and then you can cook it so that’s what I like about it.” (Fae, working 21 to <35 hours per week)	
	Functionality	“I liked how it had allergies and ingredients that you liked and you disliked and how it had meal sizes, meal servings (...) you could really, like, make it for your family...” (Fae, working 21 to <35 hours per week)	
Ongoing use			“...at least like once a week when I, because I do my grocery shopping usually once a week. So I’ll probably sit down the night before and, you know, meal plan what we’re going to have for that week.” (Kathryn, working 1 to <21 hours per week) “...I would do another week every couple of months on it when I was looking for inspiration.” (Lana, working 35+ hours per week)

### Buy-in

Early impressions of the apps appeared to be key to user buy-in and subsequent use. The alignment or fit of the app’s purpose with participants’ self-identified needs was important, as was the clarity of the purpose of the app. Trying to do too much or serve too many purposes was problematic.

The look and feel of the apps were also important for buy-in and used to judge credibility and trustworthiness. The esthetic quality subscale score of the uMARS aligned well with the interview data, with the more visual apps (ie, the recipe app, meal planning app, and barcode scanning app) scoring higher on the subscale. The same 3 apps ranked the highest for perceived credibility (Table 4).

The hedonic value of apps, or the pleasure associated with their use, played a role in app buy-in. Novelty was important for some participants. However, the lack of pleasure associated with the use of these apps was an issue, particularly for apps with little content. Of the 5 engagement subscale items of the uMARS, 2 relate to the hedonic value of apps, namely, the entertainment and interest qualities. Overall, the engagement subscale had the lowest scoring quality of the apps (Table 4), with the high-input and low-content apps again scoring the lowest on this domain.

**Table 4:** Mean (SD) uMARS scores, subjective quality score, and total score by app (n=62).

App	Value, n (%)^a^	Subscale score^b^, mean (SD)					Total uMARS^c^ score^b^, mean (SD)		Subjective quality score^b^, mean (SD)
		Engagement	Functionality	Esthetics	Credibility				
Meal planning app	35 (56.5)	3.5 (0.5)	4.2 (0.5)	4.1 (0.6)	4.0 (0.8)	3.9 (0.5)		3.0 (0.9)	
Recipe manager app	32 (51.6)	3.0 (0.7)	3.7 (0.9)	3.4 (0.6)	3.8 (0.8)	3.5 (0.6)		2.7 (1.1)	
Recipe app	29 (46.8)	3.7 (0.6)	4.2 (0.6)	4.3 (0.5)	4.1 (0.7)	4.1 (0.4)		3.1 (0.9)	
Barcode scanning app	12 (19.4)	3.6 (0.7)	4.2 (0.6)	4.0 (0.7)	4.3 (0.8)	4.0 (0.6)		3.4 (1.0)	
Family organizer app	12 (19.4)	3.4 (0.8)	3.8 (0.7)	3.8 (0.7)	3.6 (0.5)	3.6 (0.6)		2.7 (1.1)	

^a^n=4 participants completed the user version of the Mobile App Rating Scale for only one app because of a lack of use of the second app.

^b^Scores range from 1 (low quality) to 5 (high quality).

^c^uMARS: user version of the Mobile App Rating Scale.

### Use

More participants reported using the apps at least once in the first week than in the subsequent weeks (Multimedia Appendix 3). The barcode scanning app was used most frequently over the testing period, with at least 7 of the 12 participants allocated the app using it at least 2 to 4 times per week. Of the 12 users, 9 reported spending 1 to 5 minutes at a time on the app. There was a rapid drop-off in the use of the family organizer after the first week. There was also some decline in the use of the meal planning app over time; however, it was used for more than 5 minutes on each occasion by more than 22 of the 35 users.

Participants’ descriptions of the timing and context in which they used the apps led to 2 key subthemes: in-time and when-time. In-time use of apps, while planning meals, shopping, or cooking, was purposeful or planned and undertaken to achieve a task. When-time use tended to be exploratory and took place in spare moments when it was convenient to do so. This use appeared to be for information or inspiration seeking, as opposed to functional tasks.

Another subtheme of app use was the depth to which the participants used the apps. During discussions of app features, some participants described not exploring the apps deeply enough to have knowledge of their content, features, and functionality. This may have affected participants’ perception of app utility, as some even described wanting features that were already present in the apps.

### Barriers

Participants described external barriers impacting their acceptance and use of the apps and their ability to incorporate the apps as new behavioral strategies for food provision. Participants reported time scarcity as a barrier to app use, whereas others suggested that they had more important priorities than using an app or changing their food provision behavior. Existing habits such as paper-based shopping lists or tried and tested recipes were also a key barrier to participants’ willingness and ability to use the apps. Habits were described as difficult to change, and the formation of new habits, such as using the apps, was seen as challenging.

### User Experience

Participants’ experience with the apps was organized under 2 major themes: the behavioral performance of the apps and the effort associated with use. Behavioral performance encompassed the contribution apps made to the performance of food provision behaviors, whereas effort referred to their ease of use and functionality. These aspects of the apps were weighed against one another to determine app acceptability.

#### Behavioral Performance

Participants found recipe content useful in providing inspiration for meals and encouraging variety. Some felt that this inspiration led to a positive dietary change. Conversely, many felt that the recipes should be better tailored to families with young children and include practical nutrition information focused on national guidelines. The barcode scanning app was found to be helpful for food selection; however, only a relatively small sample indicated a need for such support.

The organization and efficiency aspects of the meal planning app and the recipe manager app were generally found to be positive, with participants discussing planning ahead, being prepared, and feeling organized. Participants found automation features, such as automated shopping list generation, useful. Planning was reported to reduce the last-minute decision making and shopping and increase accountability. However, for those participants who already considered themselves planners, the apps were simply described as an alternative tool to use when undertaking established behaviors. A suggestion for enhancing the efficiency of the apps with automated shopping list generation was to integrate the shopping list with internet-based shopping, allowing the completion of the process from planning to purchasing.

Support and communication features were mainly found in the family organizer app, which was not well accepted by study participants due to a perceived lack of relevance to families with young children. Regardless, around half of the sample were interested in syncing between devices so that other family members could contribute to food provision–related tasks.

#### Effort

Ease and simplicity of use were referred to in relation to the meal planning app, barcode scanning app, and recipe app. The SUS scores aligned well with this finding, with the same 3 apps scoring above 70 (median SUS score: meal planning app 78, IQR 68-88; barcode scanning app 79, IQR 56-90; and recipe app 80, IQR 58-89), indicating a passable level of usability. The recipe manager app was also deemed to be passable (median 75, IQR 54-86) despite receiving mixed reviews during the interviews. Conversely, the usability of the family organizer app was deemed poor (median 48, IQR 34-73). Participants reported in the interviews that the meal planning app, barcode scanning app, and recipe app were particularly easy to use because they were more intuitive, self-explanatory, and required very little input from the user. Participants also spoke about the accessibility and convenience of the technology and the streamlining of processes.

The functionality subtheme of effort described the functioning of particular features of the apps. Participants liked the personalization aspects of the apps, from modifying portion sizes to filtering recipes according to dietary requirements. Some participants suggested that the inclusion of recipe importing (a feature of the recipe manager app) in the meal planning app would further enhance personalization of content. Similarly, although participants liked the automatic generation of lists, they preferred those that they could personalize or modify as per their needs. A limitation reported by participants regarding the barcode scanning app was the inability to use it while doing internet-based shopping.

### Ongoing Use

Most participants reported that they would aim to use at least one of the apps periodically into the future, as required or when they had time. Those that found the apps particularly useful were clearer about their planned future use, whereas some articulated specific plans around further using the apps in different or extended ways.

## Discussion

### Principal Findings

This study aims to assess the feasibility of existing commercially available apps to support working parents in planning, purchasing, and preparing healthy family meals. The apps tested were found to enable in-time planning behavior, promoting organization and efficiency in food provision processes. The effort involved in using these apps had a key influence on acceptability and was weighed against the perceived benefits of the apps. The balance between these factors appeared to be key to the usefulness of these apps as tools to support food provision. The lack of family friendly recipes and nutrition content was a limitation to the utility of the apps in this sample of parents.

### App Utility

Organization resulting from planning features was perceived to reduce the time burden of food provision, confirming findings from a previous review of commercial apps [14]. Planning strategies for managing food provision were found to be used by working mothers experiencing time scarcity in qualitative research [33]. These same strategies have also been associated with a higher intake of vegetables and fruits in Australian women [34]. However, planning may be challenging for those with less predictable work schedules and different family structures [35] and those who are less inclined to plan [33]. Automated and streamlined planning features, such as the generation of shopping lists from meal plans and recipes, might make these apps more widely accepted and appealing even to nonplanners.

The use of planning features in time, when food provision tasks were being undertaken, suggested that food provision apps are well placed to deliver support and content in time and context [36]. Ecological momentary interventions (EMIs) support individual behavior in everyday life outside of research or clinical settings [13]. Evidence for EMI in the app-based nutrition space is limited [36,37], with the vast majority of research describing in-time dietary monitoring, assessment, and feedback [38-40]. The integration of these apps into daily life may be key to their ability to modify or support behavior. Furthermore, integration into daily life may overcome some of the engagement-related challenges experienced by app-based interventions in the past.

Inspiration was the main subtheme arising from discussions regarding apps with recipe content. Research investigating parental preferences for a food provision program targeting young children found that participants with higher income, older participants, and partnered participants were interested in creative cooking without recipes [41]. The sample in this study was similarly biased, perhaps explaining the use of recipe content for inspiration. The lack of family friendly recipes (ie, recipes that are acceptable to children and adults alike) may have limited parents’ ability to use the recipes for anything other than inspiration.

Participants aptly suggested the need for more explicit nutrition content in the form of serve-based information linked to recipes. Previous work investigating parental preferences for an eHealth family healthy lifestyle program similarly found that parents preferred more practical nutrition information such as healthy portion sizes and recipes [42]. The barcode scanning app included in this study provided nutrition-related content and was well accepted and rated well for quality and usability by users, in line with the findings of a previous review [14]. However, according to the COM-B self-assessment, the app was not widely needed in this subset, resulting in only 12 users being allocated to the app. These findings suggest that although food provision apps may be capable of supporting the behavioral performance of food provision processes, their lack of practical nutrition content delivered in a way that suits the needs of families limits their utility in addressing diet quality in the family context.

### App Acceptability

#### Engagement and Quality

Relatively few participants used the apps consistently and with the depth expected upon allocation. Barriers to buy-in and use included time scarcity and existing habits. Time scarcity is a common barrier to healthy food provision behaviors [11,12]. It is, therefore, unsurprising that time could also act as a barrier to the uptake of new digital solutions to food provision, especially when the use of such technology requires the formation of new habits [43]. Existing food provision habits are formed with repetition and practice and can be difficult to break, particularly in a stable environment [44]. Previous research has similarly found that time and habits are key barriers to food provision behaviors and the uptake of a meal planning app targeting low-income parents [45]. Aligning app use with everyday food provision tasks (such as meal planning), automation of key features, and integration with services such as internet-based shopping may alleviate the time burden of app use itself. Positioning food provision apps as tools to support the maintenance of healthy habits during times of stress or disruption may reduce the need for new habit formation.

Consistent with previous work [14,46], engagement quality was the lowest scoring uMARS subscale, particularly in those apps with minimal existing content. This is concerning, as user engagement is a major challenge to the efficacy and longevity of mHealth interventions [9]. The novelty of the apps was positive, as it is thought to play an early role in the hedonic value of technology [43]. However, familiarity with technology tends to reduce the pleasure derived from its novelty [43]. This suggests a need for other qualities to promote ongoing engagement. As the enjoyment or pleasure associated with the use of technology has been shown to be important to the usability, acceptance, and use of apps [43,47], enhancing this aspect should be a key consideration in the future. Features such as gamification can achieve this [48]. Providing an adequate onboarding experience highlighting the app’s purpose and functionality may also be helpful in promoting a better depth of engagement.

#### Usability and Functionality

Participants in this study weighed the behavioral performance of the apps against the effort required to make use of them, with effort reflecting aspects of usability and functionality. According to the consumer version of the Unified Theory of Acceptance and Use of Technology, the effort involved in technology use is thought to be a key predictor of intention to use, acceptance, and actual use of technology [43]. Furthermore, the level of effort or the process involved in using technology may be more important to women [43], who made up the majority of participants in this study. The greater acceptance of the apps requiring less user input (such as the recipe app and the barcode scanning app) suggests the need for careful consideration of the balance between effort and behavioral performance in future app development. This finding strengthens the case for automated features and integration of apps with daily life, which has been shown to be a high priority for both digital health experts and consumers in addressing the usability of health-related apps [47]. The generation of shopping lists from meal plans, entry of grocery items using a barcode scanner, and integration with internet-based shopping might improve app usability.

### Context and Need

The parents in this study identified a need for information in the form of healthy recipes and meal ideas and for ways to reduce the time burden of food provision rather than for support with food choice or food preparation skills. This may reflect the higher income and education level of the sample, which has been previously associated with greater food and nutrition knowledge, skills, and confidence [49,50]. A discrete choice experiment showed that older parents, parents with higher income, and partnered parents had a preference for meal planning and time-saving strategies, whereas younger parents, parents with lower income, and single parents preferred support with healthy cooking and nutrition [41]. Future research in households where needs are different could consider apps and app features that were less represented in this work (eg, apps focused on nutrition information and food preparation skills).

### Strengths and Limitations

Although this study does not assess dietary behavior change resulting from the use of these mobile apps, it provides early evidence to support future app development and testing. The strength of this study was its mixed methods approach, including the allocation of apps based on need and the incorporation of rich qualitative data triangulated with quantitative findings. However, despite allocating apps according to need, they were not always deemed relevant or suitable. Parents testing the family organizer app felt that it was not relevant to families of young children; therefore, the feedback regarding this app should be interpreted with caution. There were also limitations with regard to the sample population, which may limit the generalizability of the findings. The sample was typically of high socioeconomic status, which may have led to homogeneity in the results. However, efforts were made to incorporate the voices of single parents and parents of lower socioeconomic status. Irrespective of this sample bias, the vegetable, fruit, and discretionary choice intake of the study sample reflected the eating habits of the broader Australian population [51,52]. Therefore, the sample in this study would benefit from food provision–related support as much as the broader Australian population.

### Implications for Practice and Future Research

The behavior change potential of food provision apps may lie in their ability to be integrated into everyday life, promoting healthy food provision in time and context. Meal planning apps with automated planning and shopping list preparation and integration with internet-based shopping and between users may provide a nexus between dietary guidelines and healthy food provisioning and enable planning behavior in those less inclined to plan. Before more rigorous efficacy testing, future research should endeavor to strengthen the behavior change content of these apps by including features directly addressing time scarcity and parents’ need for healthy recipes and meal ideas. However, other digital tools that may be used to achieve similar goals (such as recipe websites, grocery shopping websites, and social media) are also worth considering.

### Conclusions

This study has provided insights into the role of mobile apps in supporting parents to achieve healthy food provision in a family context. Meal planning apps and features promoting organization present feasible, time-saving solutions to support healthy food provision practices. However, the time burden of app use may outweigh the time saved in the food provision process. A balance must be achieved between effort and outcome to improve the usability and usefulness of these apps. To progress in this area, attention must be paid to enhancing app automation and integration as well as recipe and nutrition content to support healthy food provision behavior in time.

## Appendix

Multimedia Appendix 1 Details of apps tested.

Multimedia Appendix 2 Mapping of apps to COM-B (Capability, Opportunity, Motivation, and Behavior) items.

Multimedia Appendix 3 Frequency and duration of app use.

## Abbreviations

COM-B: Capability, Opportunity, Motivation, and Behavior
EMI: ecological momentary intervention
NCD: noncommunicable disease
SUS: System Usability Scale
uMARS: user version of the Mobile App Rating Scale
