# Supplementary material for: Commercially Available Apps to Support Healthy Family Meals: User Testing of App Utility, Acceptability, and Engagement
Source: JMIR Mhealth Uhealth. 2021 May 7;9(5):e22990. doi: 10.2196/22990 (PMC8140382; doi:10.2196/22990)
Supplement: Multimedia Appendix 1 [file mhealth_v9i5e22990_app1.docx]

**Multimedia Appendix 1. Details of apps tested**

Mobile App Rating Scale (MARS) quality score, BCTs and key content and features of the apps selected for testing from Mauch et al., 2018

| **App** | **MARS quality score** | **Key content & features of interest** | **No of BCTs** | **BCTs linked with app content & features^a^** |
| --- | --- | --- | --- | --- |
| Meal planning app | 4.2 | Recipes – personalized based on preferences  Meal planning – automated or manual  Shopping list – automatically generated from recipe content | 7 | - 1. Goal setting (behavior)   2.3 Self-monitoring of behavior  3.1 Social support (unspecified)  4.1 Instruction on how to perform the behavior  5.3 Information about social & environmental consequences  5.6 Information about emotional consequences  12.5 Adding objects to the environment |
| Recipe manager app | 3.7 | Recipe storage – automatic population from online or manual entry  Meal planning – manual  Shopping list – automatically generated from recipe content | 4 | 1.1 Goal setting (behavior)  1.4 Action planning  4.1 Instruction on how to perform the behavior  12.5 Adding objects to the environment |
| Recipe app | 4.2 | Recipes – text, photo and video  Food preparation skills – text, photo and video | 4 | 4.1 Instruction on how to perform the behavior  6.1 Demonstration of the behavior  6.2 Social comparison  12.5 Adding objects to the environment |
| Barcode scanning app | 3.9 | Product specific nutrition information – generated via barcode scanner | 2 | 8.2 Behavioral substitution  12.5 Adding objects to the environment |
| Family organizer app | 4.2 | Shopping list – synced between users  Family calendar – personalized tasks and reminders | 10 | 1.1 Goal setting (behavior)  1.4 Action planning  2.3 Self-monitoring of behavior  3.1 Social support (unspecified)  5.3 Information about social & environmental consequences  7.1 Prompts/cues  10.3 Non-specific reward  10.6 Non-specific incentive  12.5 Adding objects to the environment  14.1 Behavior cost |

a BCTs with numbers according to the Behavior Change Technique Taxonomy Version 1
