# Supplementary material for: Commercially Available Apps to Support Healthy Family Meals: User Testing of App Utility, Acceptability, and Engagement
Source: JMIR Mhealth Uhealth. 2021 May 7;9(5):e22990. doi: 10.2196/22990 (PMC8140382; doi:10.2196/22990)
Supplement: Multimedia Appendix 2 [file mhealth_v9i5e22990_app2.docx]

**Multimedia Appendix 2. Mapping of apps to Capability, Opportunity, Motivation, and Behavior items.**

COM-B self-evaluation items mapped to corresponding apps and app content/features

| **What do you think it would take for you to provide healthier meals for your family?**  ***I would need to:*** | | **App** | **Relevant content / feature** |
| --- | --- | --- | --- |
| Capability | Have better food preparation and/or cooking skills | Recipe app | Recipe and food preparation skills content |
|  | Learn how to choose healthy food at the supermarket | Barcode scanning app | Product specific nutrition information |
|  | Learn how to plan healthy meals | Meal planning app | Automated meal planning based on preferences |
| Opportunity | Have more time to plan, buy and prepare healthy meals | Meal planning app | Automated meal planning and shopping list generation |
|  | Have more healthy recipes and meal ideas | Recipe app | Recipe content |
|  | Have guidance in choosing healthy food/meals | Barcode scanning app | Product specific nutrition information |
|  | Have a better way of planning and recording meals and groceries for the coming week | Recipe manager app | Recipe storage, meal planning, shopping list |
|  | Have more support or help from my partner/family | Family organiser app | Shared calendar & shopping lists |
|  | Have more reminders to plan, shop or cook | Family organiser app | Task & calendar event reminders |
| Motivation | Have clear goals or plans toward preparing healthy meals | Recipe manager app | Manual meal planning |
