# Supplementary material for: Commercially Available Apps to Support Healthy Family Meals: User Testing of App Utility, Acceptability, and Engagement
Source: JMIR Mhealth Uhealth. 2021 May 7;9(5):e22990. doi: 10.2196/22990 (PMC8140382; doi:10.2196/22990)
Supplement: Multimedia Appendix 3 [file mhealth_v9i5e22990_app3.docx]

**Multimedia Appendix 3. Frequency and duration of app use.**

Self-reported frequency of app use over the 4-week app testing period, by app, and across the total sample (n=62)

| **Apps** | **N** | **Week** | **Didn’t use the app** | **Once** | **2-4 times** | **5 or more times** |
| --- | --- | --- | --- | --- | --- | --- |
|  |  |  | **n (%)** | **n (%)** | **n (%)** | **n (%)** |
| Meal planning app | 35 | 1 | 5 (14) | 15 (43) | 14 (40) | 1 (3) |
|  |  | 2 | 11 (31) | 16 (46) | 8 (22) | 0 (0) |
|  |  | 3 | 15 (43) | 7 (20) | 12 (34) | 1 (3) |
|  |  | 4 | 19 (54) | 7 (20) | 9 (25) | 0 (0) |
| Recipe manager app | 32 | 1 | 3 (9) | 16 (50) | 13 (41) | 0 (0) |
|  |  | 2 | 11 (34) | 9 (28) | 12 (38) | 0 (0) |
|  |  | 3 | 11 (34) | 10 (31) | 11 (34) | 0 (0) |
|  |  | 4 | 16 (50) | 10 (31) | 5 (16) | 1 (3) |
| Recipe app | 29 | 1 | 4 (14) | 9 (31) | 13 (45) | 3 (10) |
|  |  | 2 | 7 (24) | 11 (38) | 10 (35) | 1 (3) |
|  |  | 3 | 9 (31) | 12 (41) | 6 (21) | 2 (7) |
|  |  | 4 | 12 (41) | 7 (24) | 9 (31) | 1 (3) |
| Barcode scanner app | 12 | 1 | 2 (17) | 3 (25) | 3 (25) | 4 (33) |
|  |  | 2 | 1 (8) | 4 (33) | 6 (50) | 1 (8) |
|  |  | 3 | 2 (17) | 3 (25) | 4 (33) | 3 (25) |
|  |  | 4 | 3 (25) | 1 (8) | 4 (33) | 4 (33) |
| Family organiser app | 12 | 1 | 0 (0) | 5 (42) | 4 (33) | 3 (25) |
|  |  | 2 | 7 (58) | 1 (8) | 3 (25) | 1 (8) |
|  |  | 3 | 6 (50) | 2 (17) | 3 (25) | 1 (8) |
|  |  | 4 | 7 (58) | 2 (17) | 2 (17) | 1 (8) |

Table 2: Self-reported duration of use of apps on each interaction during the 4-week testing period, by app and across the total sample (n=62)

| **App** | **N** | **Less than 1 minute** | **1 to 5 minutes** | **More than 5 minutes** |
| --- | --- | --- | --- | --- |
|  |  | **n (%)** | **n (%)** | **n (%)** |
| Meal planning app | 35 | 2 (6) | 11 (31) | 22 (63) |
| Recipe manager app | 32 | 1 (3) | 16 (50) | 15 (47) |
| Recipe app | 29 | 0 (0) | 14 (48) | 15 (52) |
| Barcode scanner app | 12 | 0 (0) | 9 (75) | 3 (25) |
| Family organiser app | 12 | 1 (8) | 5 (42) | 6 (50) |
